# A randomized, controlled, crossover trial to assess the acute appetitive and metabolic effects of sausage and egg-based convenience breakfast meals in overweight premenopausal women

**DOI:** 10.1186/s12937-015-0002-7

**Published:** 2015-02-10

**Authors:** Tia M Rains, Heather J Leidy, Kristen D Sanoshy, Andrea L Lawless, Kevin C Maki

**Affiliations:** 1 SALTT, LLC, 801 Ouilmette Ln, Wilmette, IL 60091 USA; 2 Department of Nutrition and Exercise Physiology, University of Missouri, 307 Gwynn Hall, Columbia, MO 65201 USA; 3 Biofortis Clinical Research, 211 E. Lake St, Addison, IL 60101 USA; 4 Midwest Center for Metabolic & Cardiovascular Research, 489 Taft Ave., Suite 202, Glen Ellyn, IL 60137 USA

**Keywords:** Hunger, Fullness, Appetite, Protein, Glycemic control

## Abstract

**Background:**

Dietary protein at breakfast has been shown to enhance satiety and reduce subsequent energy intake more so than carbohydrate or fat. However, relatively few studies have assessed substitution of protein for carbohydrate on indicators of appetite and glucose homeostasis simultaneously.

**Methods:**

The acute appetitive and metabolic effects of commercially-prepared sausage and egg-based breakfast meals at two different protein levels (30 g and 39 g/serving), vs. a low-protein pancake breakfast (3 g protein) and no breakfast (water), were examined in premenopausal women (N = 35; age 32.5 ± 1.6 yr; BMI 24.8 ± 0.5 kg/m^2^). Test products provided ~280 kcal/serving and similar fat (12–14 g) and fiber contents (0–1 g). Visual Analog Scale ratings for appetite (hunger, fullness, prospective consumption, desire to eat) and repeated blood sampling for plasma glucose and insulin concentrations were assessed throughout each test day. Energy intake was recorded at an *ad libitum* lunch meal at 240 min.

**Results:**

Results showed increased satiety ratings for both the 30 and 39 g protein meals vs. the low-protein and no breakfast conditions (*p* < 0.001 for all). Postprandial glucose and insulin excursions were lower following the 30 g and 39 g protein conditions vs. the low-protein condition, with smaller responses following the 39 g vs. 30 g protein condition (*p* < 0.05 for all). Energy intake at lunch was significantly less (*p* < 0.001) following the 39 g protein meal (692 kcal) vs. the low-protein and no breakfast conditions (789 and 810 kcal, respectively). Total energy intake from the test condition + lunch was higher (*p* < 0.01) for the 30 and 39 g meals (982 and 983 kcal, respectively) vs. no breakfast (810 kcal), and less than the low protein breakfast (1064 kcal; *p* < 0.01 vs. 39 g condition only).

**Conclusions:**

Results suggest that convenience meals providing 30 or 39 g protein/serving produce greater appetite control, lower postprandial glycemia and insulinemia, and reduced subsequent intake at lunch relative to a low-protein control, or no breakfast.

**Trial registration:**

NCT01713114

## Background

Protein is generally regarded to be more satiating than an equivalent amount of digestible carbohydrate or fat [1-4]. This may be particularly true when protein is consumed at breakfast versus later in the day, as studies that have fed protein at lunch or dinner have shown more variable results [5]. Acute intervention studies have shown that protein-rich breakfast meals reduce appetite and increase satiety throughout the morning relative to moderate or low-protein breakfast meals [6-8]. Protein-rich breakfast meals have also been shown to reduce energy intake at a subsequent *ad libitum* lunch meal [6,7,9-11]. Such effects have been demonstrated at protein intakes ≥20 g, and most consistently at intakes ≥30 g of protein per meal [6,7,9-12].

The effects of a protein-rich breakfast may extend beyond the immediate postprandial period. Several investigators have reported reduced energy intakes over the 24 hour period following egg-based breakfast meals rich in protein [7,10], although not all studies have produced similar results [13]. Leidy et al. [12] found that higher protein intake at breakfast (35 vs. 13 g) was associated not only with greater satiety and less hunger throughout the morning, but also with reduced energy intake from snacks in the evening hours, particularly high-fat snacks.

Limited data exist regarding postprandial glucose and insulin excursions and their relationships to appetitive responses following high vs. lower protein meals. In addition to the satiety properties of dietary protein, the consumption of higher protein meals has been proposed to improve glucose homeostasis. Several meta-analyses from long-term, higher protein, weight loss and/or weight maintenance diets report reductions in glycated hemoglobin and/or fasting insulin concentrations with higher vs. normal protein diets [14,15]. Since larger postprandial glucose elevations have been shown to be associated with an increased risk for the development of type 2 diabetes mellitus [16] and cardiovascular disease [17], it is of interest to identify dietary strategies, such as higher protein intake at breakfast, that might improve glucose homeostasis through the reductions in these responses.

Average dietary protein intake in the US is adequate based on current recommendations [18]. However, data from the National Health and Nutrition Examination Survey (NHANES) survey suggest that the majority of dietary protein is consumed at dinner, with protein intakes at breakfast averaging ~10 g in women and 15 g in men, well below levels shown to favorably affect appetite and metabolism [19]. In addition, approximately 20% of US men and women do not consume breakfast [20]. In an analysis of NHANES data, our group found that higher protein intake at breakfast was inversely associated with energy intake at lunch, and higher non-protein intake at breakfast was positively associated with energy intake at lunch [19].

Given ready-to-eat cereals and other foods requiring little preparation are frequently consumed at breakfast, convenient breakfast options high in protein would be potentially beneficial for individuals interested in reducing morning hunger and energy intake later in the day as well as glycemic excursions. The present study was undertaken to evaluate the effects of consuming two higher-protein sausage and egg-based frozen convenience breakfast meals, providing 30 or 39 g of protein, compared with a lower-protein, higher carbohydrate frozen convenience breakfast meal (pancakes and syrup, 3 g protein), and breakfast skipping, on appetite ratings, postprandial glycemic and insulinemic responses, and *ad libitum* energy intake at a lunch meal in normal weight to overweight, premenopausal women.

## Methods

### Design

This was a randomized, controlled, crossover study conducted at Biofortis Clinical Research (Addison, IL) according to Good Clinical Practice Guidelines, the Declaration of Helsinki (2000), and the United States 21 Code of Federal Regulations. An institutional review board (Quorum Review IRB, Seattle, WA) approved the protocol before initiation of the study and subjects provided written informed consent before any study procedures were performed. The study included 1 screening visit and 4 test visits, each separated by at least 5 days.

### Participants

Healthy premenopausal women aged 18 to 55 y, each with a body mass index (BMI) 18.5 to 29.9 kg/m^2^ and who were regular consumers of breakfast and lunch (≥5 days/week), were recruited to participate. Subjects were excluded if they were self-defined smokers; reported a recent weight change of ±2.7 kg; had history of surgical intervention for the treatment of obesity; used weight loss medications, supplements, programs, or meal replacement products; used medications or dietary supplements likely to markedly affect taste, smell, or appetite; or scored >11 on a dietary restraint scale [21]. Subjects with a history of cardiac, renal, hepatic, endocrine, pulmonary, biliary, pancreatic, gastrointestinal or neurologic disorders, or cancer (in the last 2 years); known sensitivity, allergy, or taste aversion to any of the ingredients in the study products; a history of eating disorders or alcohol abuse; and use of medications known to influence carbohydrate metabolism were also excluded from the study. Subjects who developed symptoms of active infection during the study period (e.g., upper respiratory infection) or reported antibiotic use were allowed to continue only after symptoms had been resolved and antibiotic use had been discontinued at least 5 days prior to testing.

In total, 34 women participated in the study (Table 1). Test visits were scheduled during the follicular phase of each woman’s menstrual cycle, defined as days 1 to 14, where day 1 is the first day of menses. Participants were instructed to contact the clinic once menses began to schedule their test visits. Additional study instructions included avoidance of vigorous physical activity, consumption of alcoholic beverages, and maintenance of habitual caffeine intake within 24 hours of each test visit. Subjects were also dispensed a food record prior to their first test visit, which was copied and dispensed to each participant with instructions to replicate the same food and beverage intakes to the best of their ability after 1400 hour the day prior to subsequent test visits.

**Table 1:** **Subject characteristics**

Characteristic	Efficacy evaluable population
	**N (%)**
Female	34 (100)
Race/Ethnicity	
Non-Hispanic white	20 (58.8)
African American	7 (20.6)
Other	7 (20.6)
	**Mean (SEM)**
Age (y)	32.2 (1.6)
Weight (kg)	66.9 (1.5)
Body mass index (kg/m^2^)	24.9 (0.5)
Restraint score (Arbitrary units)	6.5 (0.6)

### Procedures

**Figure 1:**
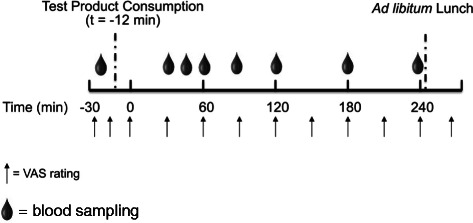
**Test visit flow diagram.**

On test days, subjects reported to the clinic (~0800 hour) following an overnight fast (10–12 hour). An intravenous (IV) catheter was inserted for collection of venous blood. At approximately t = −60 min, subjects were offered 6 oz of water, a caffeinated, non-caloric cola, caffeinated coffee, or tea with non-caloric sweetener. The beverage of their choice was replicated at subsequent test visits. At t = −12 min, subjects were administered one of three study products with 175 g of water or water only, and instructed to consume approximately one-third of the study product and water every 4 min (Figure 1). Participants were instructed to consume the study product and water in their entirety. Participants were provided with 500 g of water and allowed to drink *ad libitum* throughout the remainder of the visit. Actual water intake was recorded.

Validated Visual Analog Scale (VAS) ratings [22] for appetite sensations (hunger, fullness, desire to eat, and prospective food consumption) were completed prior to study product intake (t = −25 and −15 min) and at 30 min intervals until 240 min. VAS ratings were recorded on a 100-mm horizontal line anchored by “not at all” to “extremely” in response to questions worded as “How strong is your feeling of”. Palatability was assessed by a 9-point scale with the anchors “dislike extremely” and “like extremely” immediately following the last bite of study product.

Blood draws were performed at specific times (Figure 1). Seven blood samples (4 ml/sample; 28 ml/testing day) were collected throughout each testing day. The samples were collected in test tubes containing ethylenediaminetetraacetic acid. Within 10 min of collection, the samples were centrifuged at −4 °C for 10 min. The plasma was separated and stored in microcentrifuge tubes at −80 °C for future analysis. Plasma glucose was measured through an in-house glucose oxidase assay (Thermo Fisher Scientific, USA). Plasma insulin was measured using the Milliplex MAP magnetic bead-based multi-analyte, metabolic panel (Millipore, St. Charles, MO) and Magpix Luminex technologies (Luminex Corporation, Austin, TX).

An *ad libitum* lunch consisting of tortellini and sauce was provided to participants following the last blood collection. Subjects were allowed 25 min for lunch and instructed to eat until “comfortably full”. Food was weighed to the nearest gram prior to and following consumption. Subjects were provided with a standard amount of water during lunch and the quantity of water consumed was recorded. A final VAS rating was recorded following lunch at t = 270 min.

### Test products

Test products included two commercially-prepared, frozen breakfast meals consisting of egg white, poultry sausage, potatoes, and cheese at two different protein levels [30 g and 39 g/serving; Hillshire Brands); a low protein (LP) meal consisting of mini-pancakes (Eggo®, Kellogg’s), syrup (Aunt Jemima®, The Quaker Oats Company), butter (Land O’Lakes); and no breakfast (NB; water only) (Table 2). All test products were prepared according to manufacturer instructions. Each meal was similar in energy, total fat, and fiber contents (except water only condition), but differed in total weight (77 g, 189 g, and 178 g for the LP, 30 g and 39 g bowls, respectively).

**Table 2:** **Test meal characteristics** ^**1**^

	NB	LP	30 g Pro	39 g Pro
Energy (kcal)	0	288	280	294
Protein (g)	0	3	30	39
Carbohydrate (g)	0	44	13	3
Total fat (g)	0	11	12	14
Fiber (g)	0	1	0	0
Palatability (au)^2^	--	7 (7, 8)	7 (6, 8)	6 (4, 7)

^1^NB, no breakfast; LP, low protein; Pro, protein.

^2^Palatability was assessed by a 9-point scale with 1 = “dislike extremely” and 9 = “like extremely.” Median values (interquartile limits) are presented.

### Statistical analyses – VAS responses and subsequent food intake

Statistical analyses were conducted using SAS version 9.1.3 (SAS Institute, Cary, NC). An evaluable sample size of 33 subjects was expected to provide 85% power to detect a difference of 2400 mm * min in the net incremental area under the curve (niAUC) for postprandial VAS scores between treatment conditions, assuming a standard deviation of 3816 mm * min (based on prior work by our group), for an effect size of 62%. A nominal alpha of 0.017 was used for sample size calculations to account for three primary comparisons between the three energy-containing meal conditions [23].

VAS rating niAUCs were calculated by applying the trapezoidal rule for both positive and negative increments from pre-meal (average of timepoints, nominal time 0 min was calculated as the average of the values at −25 and −15 min) to 240 min, whereas total AUC was calculated for the plasma glucose and insulin concentrations throughout the 240 min [24]. Repeated measures analysis of variance (ANOVA) was used to assess differences between test conditions for continuous outcome variables. Initial models contained terms for treatment condition, sequence, period, age and baseline BMI, with subject included as a random effect. Models were reduced in a stepwise manner until only significant terms or treatment condition remained in the model. The Shapiro-Wilk test was used to assess normality of the distribution of residuals in the analysis models. The normality assumption was rejected for palatability, energy intake at lunch, and preload plus energy intake, therefore rank transformations were employed and the models were rerun using the transformed data. When treatment condition main effects were detected, pairwise comparisons between conditions were conducted using Tukey’s adjustment for multiple comparisons. Sensitivity analyses were conducted using the overall liking palatability score as a covariate in models for appetite ratings and energy intake at lunch with the no breakfast condition excluded.

For the appetite and food intake outcomes, the primary efficacy analysis was completed on an efficacy evaluable (EE) sample that included all subjects who consumed the 39 g protein breakfast and at least one other condition. A secondary analysis was completed on a per protocol (PP) sample, a subset of the EE population excluding those subjects who had significant protocol deviations (e.g., use of an excluded medication, illness that caused rescheduling of visits) during the treatment period. Subjects in the PP population completed all test visits. All decisions were made prior to breaking the treatment code or locking the database by people unaware of the order of treatments. Results were similar for the EE and PP analyses; therefore, outcomes are presented only for the EE sample. However, for the glucose and insulin measures, only those completing all test conditions were included and thus a PP analysis was performed.

## Results

### Palatability

Ratings for appearance, texture, flavor, and overall liking were higher (more palatable) for the LP and 30 g protein conditions versus the 39 g protein conditions (*p* < 0.02 for all). There were no differences between the 30 g and LP conditions. Despite the fact that there were differences, there were no statistically significant associations between overall liking and any of the appetitive ratings or energy intake at lunch. Similarly, when palatability rating was included as a covariate in a subset analysis that excluded the no breakfast condition, palatability rating was did not significantly reduce the unexplained variance.

### Appetite sensation ratings

**Figure 2:**
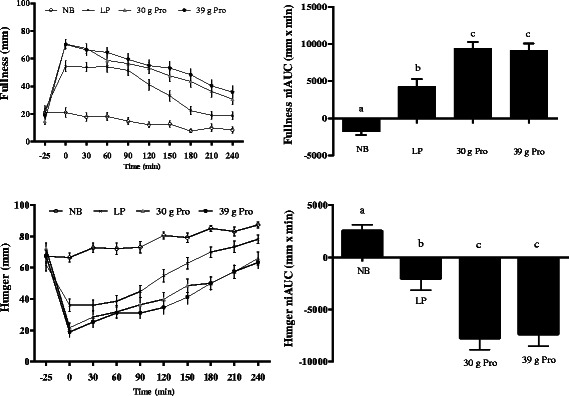
**Appetite VAS ratings (hunger and fullness) at each timepoint and niAUC values for each condition.** Data are presented as mean ± SEM. Different letters indicate differences between conditions (p < 0.0001). Pairwise comparisons between conditions were conducted using Tukey’s adjustment for multiple comparisons.

**Figure 3:**
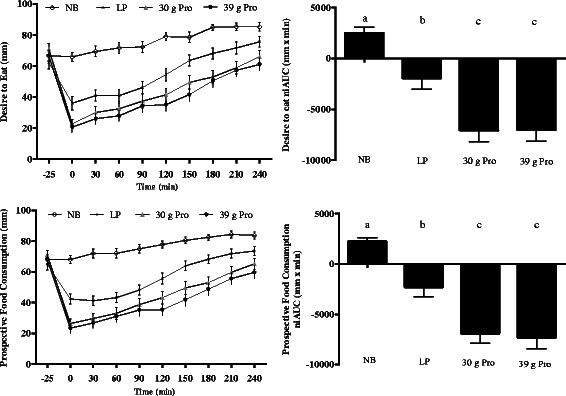
**Appetite VAS ratings at each timepoint (left) and niAUC values (right) for each condition.** Data are for desire to eat and prospective food consumption. Data are presented as mean ± SEM. Different letters indicate differences between conditions (p < 0.0001). Pairwise comparisons between conditions were conducted using Tukey’s adjustment for multiple comparisons.

Results for hunger, fullness, desire to eat, and prospective food consumption are depicted in the line graphs (individual timepoints) and bar graphs (niAUC) in Figures 2 and 3. For each appetite response, there was a main effect of treatment condition (*p* < 0.001). Pairwise comparisons showed that both the 30 g and 39 g protein conditions led to greater appetite control and satiety based on the niAUC values (*p* < 0.001 for all) compared to the NB and LP breakfast. The LP condition also produced greater ratings for satiety and reduced hunger relative to the no breakfast condition (*p* < 0.01 for all). There were no differences between the 30 and 39 g conditions for any appetite sensation rating. An exploratory analysis was conducted to compare treatment conditions for appetite sensation ratings at the 240 min timepoint immediately prior to the lunch meal. Results are summarized in Table 3. All treatments except the 30 g and 39 g protein conditions differed significantly for ratings of hunger, fullness and desire to eat (p < 0.05), with the 30 g and 39 g protein conditions producing the lowest ratings for hunger and desire to eat and the highest ratings for fullness. Prospective food consumption ratings did not differ significantly between the LP and 30 g protein conditions (p = 0.075), as well as between the 30 g and 39 g protein conditions (p = 0.587), but each of these conditions produced significantly lower ratings compared to NB (all p < 0.05).

**Table 3:** **Appetite sensation ratings at 240 min by breakfast meal condition** ^**1**^

	NB	LP	30 g Pro	39 g Pro	p-value
	Median (interquartile limits)				
Hunger (mm)	91 (79, 96)^a^	81 (70, 91)^b^	71 (50, 84)^c^	69 (49, 80)^c^	<0.0001
Fullness (mm)	3 (1, 12)^a^	15 (9, 24)^b^	26 (10, 54)^c^	33 (14, 59)^c^	<0.0001
Desire to eat (mm)	89 (81, 95)^a^	76 (69, 92)^b^	69 (47, 83)^c^	69 (39, 80)^c^	<0.0001
Prospective food consumption (mm)	85 (77, 93)^a^	77 (64, 84)^b^	67 (50, 82)^b,c^	69 (40, 79)^c^	<0.0001

^1^NB, no breakfast; LP, low protein; Pro, protein.

^a,b,c^Different superscripted letters denote statistically significant differences (p < 0.05).

### Metabolic responses

**Figure 4:**
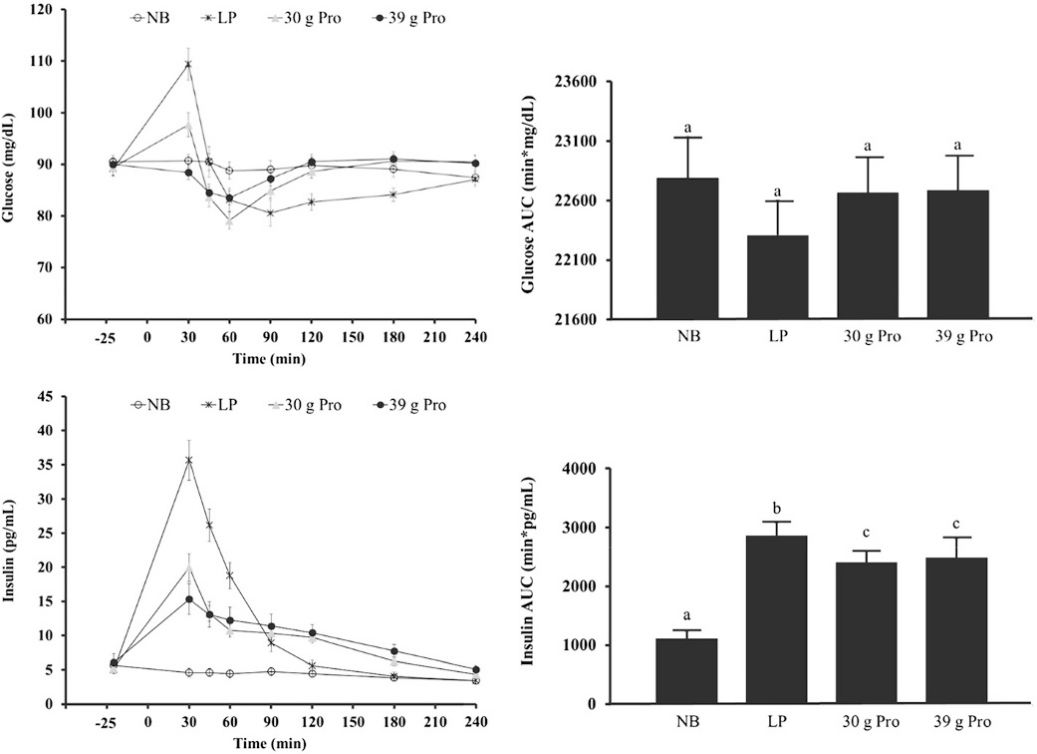
**Plasma glucose and insulin at each timepoint (left) and AUC values (right) for each condition.** Data are presented as mean ± SEM. Different letters indicate differences between conditions (p < 0.0001). Pairwise comparisons between conditions were conducted using Tukey’s adjustment for multiple comparisons.

Results for plasma glucose and insulin are depicted in the line graphs (individual timepoints) and bar graphs (AUC) in Figure 4. No main effect of treatment condition was detected for the glucose AUC response. However, a main effect of treatment condition was detected for glucose peak (*p <* 0.001) and the postprandial change in glucose (*p <* 0.001). Pairwise comparisons showed that both the NB and the 39 g protein conditions led to a lower glucose peak (both, 96 ± 1 mg/dL; *p* < 0.05 for both) compared to the LP (112 ± 3 mg/dL) and the 30 g protein (101 ± 2 mg/dl) conditions, while the 30 g protein condition led to a lower glucose peak (*p* < 0.001) compared to the LP condition. Additionally, pairwise comparisons showed that both the NB and the 39 g protein condition led to a smaller postprandial glucose change from pre-breakfast (−14 ± 1 mg/dL and −16 ± 1 mg/dL, respectively; *p* < 0.001 for both) compared to the LP (−41 ± 2 mg/dL) and the 30 g protein condition (−23 ± 2 mg/dL), while the 30 g protein condition led to a smaller postprandial glucose change (*p* < 0.001) vs. the LP condition. Lastly, the 39 g protein condition led to a smaller postprandial change in glucose (*p <* 0.05) compared to the NB condition.

Main effects of treatment condition were detected for the insulin AUC response, peak insulin, and postprandial changes (*p <* 0.001 for all). Pairwise comparisons showed that the NB condition led to lower insulin AUC (*p <* 0.001 for all) compared to all other breakfast conditions. The 30 g and 39 g protein conditions led to lower insulin AUC (*p <* 0.05 for both) vs. the LP breakfast with no differences between the 30 g and 39 g protein conditions. The NB condition led to a lower insulin peak (7 ± 1 pg/mL; *p <* 0.001 for all) compared to all breakfast conditions (i.e., low-protein (pancakes): 37 ± 3 pg/ml; 30 g protein: 20 ± 2 pg/mL; and 39 g protein: 16 ± 2 pg/mL). The 39 g protein condition led to a lower insulin peak (*p <* 0.05 for both) compared to the LP and the 30 g protein conditions. The 30 g protein condition led to a lower insulin peak (*p <* 0.001) vs. LP condition. Lastly, the NB condition led to the smallest postprandial change from pre-breakfast in insulin (−4 ± 1 pg/mL; *p <* 0.001 for all) compared to all breakfast meals (i.e. LP: −34 ± 3 pg/ml; 30 g protein: −17 ± 2 pg/mL; and 39 g protein: −11 ± 2 pg/mL. The 39 g protein condition led to a smaller postprandial change in insulin (*p <* 0.01 for both) compared to the LP and the 30 g protein conditions. The 30 g protein condition led to a smaller postprandial change in insulin (*p <* 0.001) vs. the LP condition.

### Lunch energy intake

**Figure 5:**
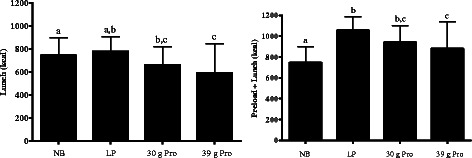
**Lunch (left) and total (preload + lunch; right) energy intake following each condition (t = 240 min).** Data are presented as median (75th percentile). Different letters indicate differences between conditions (p < 0.05, note - energy intake at lunch for the 30 g Pro breakfast vs. LP breakfast p = 0.053). Pairwise comparisons between conditions were conducted using Tukey’s adjustment for multiple comparisons.

Median energy intake at the lunch meal was reduced by approximately 15% following the 30 g and 39 g protein conditions versus the NB condition (*p* ≤ 0.03; Figure 5). Compared to the LP breakfast, energy intake was lower following the 39 g protein condition (*p* < 0.001), and was lower with the 30 g protein breakfast, but this result was only marginally statistically significant (p = 0.053). There was no significant difference in energy intake between the LP condition and NB condition. Median total energy intake from the test condition + lunch meal was higher for all meals versus the no breakfast condition (*p* < 0.001). Compared to the LP meal, total energy intake was lower after the 39 g protein meal (*p* < 0.01). There were no differences between the 30 and 39 g conditions for energy intake at lunch or preload + energy intake at lunch.

## Discussion

The results of this study showed that consumption of protein-rich convenience breakfast meals led to reductions in perceived hunger, increased satiety, and reductions in postprandial glucose and insulin excursions compared to a low-protein meal in normal to overweight, premenopausal women. The protein-rich breakfast meals also resulted in reduced energy intake at the *ad libitum* lunch meal, although results only reached significance for the 39 g protein meal. Thus, consumption of a high protein, sausage and egg-based, ready-to-heat meal may be an option for facilitating satiety throughout the morning, reducing postprandial glycemic and insulinemic excursions, and moderating energy intake at lunch.

The satiety and food intake findings are similar to results reported by other investigators in acute intervention trials using breakfasts based on solid food sources of protein. Eggs and meat as sources of protein, in particular, have been associated with greater perceived satiety and/or improved glycemic control in several studies [1,6,7,10,12,25,26]. For example, Ratliff et al. [10] compared the effects of an egg-based breakfast to a bagel-based breakfast in a group of healthy men. Similar to the results of the present study, perceived hunger, glucose, insulin, and energy intake at the lunch meal were all reduced following the higher protein breakfast. However, to the best of our knowledge, very few prior studies have evaluated the appetitive effects of protein-based, frozen convenience meals. Such meals serve as an easy to prepare option, compared to other high-protein foods traditionally consumed at breakfast in the U.S. (e.g., fresh breakfast meats, fresh eggs), which require a greater degree of preparation. This is further supported by data indicating that a primary barrier associated with breakfast is the lack of availability and convenience [27].

The present work included two levels of protein served as part of a commonly consumed breakfast meal. There were minimal differences in responses between the protein-containing meals, suggesting that both protein levels were sufficient to elicit a greater satiety response and to reduce postprandial glycemic and insulinemic excursions compared with the low-protein meal. The differences in glucose and insulin responses elicited by the test meals were expected because a substitution of protein for carbohydrate was employed, thus reducing the available carbohydrate load. Nevertheless, chronic reduction of dietary carbohydrate has been demonstrated to produce notable metabolic effects, including lowering the circulating concentration of triglycerides, reducing blood pressure, and lessening the demand for insulin production by the pancreatic beta-cells [28]. Moreover, ingestion of protein at a meal tends to increase insulin secretion without significantly increasing the plasma glucose concentration (compared to water ingestion) in the postprandial period [29]. Lastly, elevations in plasma insulin and glucose have been shown to reduce appetitive sensations [30,31]. Thus, it is notable that the appetitive effects observed with higher protein and lower carbohydrate were present despite lower insulin and glucose concentrations.

Other investigations utilizing beverages or semi-liquid applications (e.g., yogurt or custard) to evaluate the satiating properties of protein have shown similar effects to those observed in the present study [32-36]. However, such vehicles are not among the top breakfast choices in the U.S. [27]. Further, the present work included overweight and normal weight women, a group more likely to engage in strategies to reduce body weight than their male counterparts [37]. Results from several long-term intervention trials have provided evidence that higher protein, reduced carbohydrate diets may help to enhance weight loss and/or maintain lean body mass during weight loss [14,15,38-40]. Increased satiation, and therefore better adherence to caloric restriction, is one potential mechanism by which high protein diets may facilitate weight loss. High protein, easy to prepare breakfast options with greater satiating potential would likely facilitate the consumption of calorically-restricted, protein-rich diets.

The mechanisms whereby dietary protein promotes satiety are not completely understood. It has been hypothesized that a high-protein meal may modulate the post-absorptive release of hormones and neurochemicals in the gastrointestinal tract that down-regulate appetite [41]. In particular, consumption of high-protein meals has been shown to decrease levels of the hunger-stimulating hormone ghrelin and/or promote the increase in the satiety-stimulating hormones peptide YY (PYY) and glucagon-like peptide-1 (GLP-1), resulting in increased perceptions of satiety [8,9,26,42,43]. Unfortunately, data for ghrelin, PYY, and GLP-1 for the present study are not available. A majority of studies that have assessed the relationship between postprandial insulin levels and appetite sensations have suggested that insulin has an acute effect to suppress appetite [30,44], although conflicting results have been reported [45,46]. Intracerebroventricular or systemic injections of insulin suppress food intake in a dose-dependent manner in animal models, suggesting a direct involvement in satiety [47]. It is therefore notable that appetite ratings were reduced with the high protein conditions compared to the low protein (higher carbohydrate) breakfast, despite lower insulin (and glucose) responses. Some investigators have suggested that increased thermogenesis following consumption of high-protein meals, as well as changes in substrate oxidation may influence appetitive signals that affect food intake [48,49]. Additional studies will be needed to more fully define the mechanisms responsible for the effects of substituting protein for carbohydrate observed in the present study. It is also suggested that additional research is needed to assess the substitution of protein for dietary fat. Future research of interest would also include studies in which protein is substituted for fat, since the current study cannot separate the effects of increasing the protein content of the study breakfast meals from those of reducing the carbohydrate content.

Both buffet lunch and uniform food models, such as the tortellini and sauce lunch used in the present study, have been used extensively in appetite research [50]. A uniform food model was used in the present investigation because the main objective was related to energy intake rather than food selection. Thus, a limitation of the study is that possible differences in food or macronutrient preferences at lunch could not be assessed.

Another limitation is the short-term nature of the measurement period. It is possible that appetitive sensations or energy intake would increase later in the day to compensate, or even overcompensate, for reductions in energy intake observed at the lunch meal. However, results from Leidy et al.[12] suggest that this may not be the case, as total daily energy intake was reduced when a high-protein breakfast was consumed, compared with a lower protein breakfast meal. Notably, in that trial, a 7-day period of acclimation was employed for both breakfast conditions, suggesting that the effects of higher protein breakfasts on appetite do not dissipate, at least after several days of consumption.

The possibility cannot be ruled out that expectations surrounding each breakfast condition and differences in the sensory characteristics influenced the results. The present trial evaluated commercially-available breakfast options, therefore matching the sensory properties of the test conditions was not possible. However, all products were considered to be relatively well-liked and there were only measurable differences in the palatability of the highest protein condition relative to the low protein and 30 g protein conditions. Inclusion of the overall palatability rating as a covariate in sensitivity analyses for appetite ratings and energy intake at lunch did not significantly reduce the unexplained variance, suggesting that differences in palatability were unlikely to have materially influenced the results.

Differences in the physical characteristics of the study products may have also influenced orogastric transit time. In an effort to control this to the degree possible, subjects were instructed to eat a portion of each condition at specific intervals over a 12-minute period (approximately one-third during each of three 4-min periods). However, we cannot rule out the possibility that differences in consumption patterns (e.g., chewing) or orogastric transit time influenced our findings. Further, the weight and volume of the products differed. Such characteristics have been previously shown to affect perceived satiety, however our objective was to evaluate conventional breakfast options at an equivalent caloric level, and as such, differences in weight and volume were unavoidable due to the nature of the breakfast meals [51].

## Conclusion

In conclusion, the results of the present investigation suggest that sausage/egg convenience meals providing 30 g or 39 g protein per serving produce greater appetite control, reduce postprandial glycemic and insulinemic responses, and lower energy intake at lunch relative to a lower-protein, higher carbohydrate, breakfast meal.
